# METTL3-mediated m6A modification of SIRT1 mRNA inhibits progression of endometriosis by cellular senescence enhancing

**DOI:** 10.1186/s12967-023-04209-0

**Published:** 2023-06-23

**Authors:** Xiaotong Wang, Jing Wang, Xibo Zhao, Han Wu, Jixin Li, Yan Cheng, Qiuyan Guo, Xuejiao Cao, Tian Liang, Liyuan Sun, Guangmei Zhang

**Affiliations:** Department of Gynaecology, The First Affiliated Hospital of Harbin Medical University, Harbin, China

**Keywords:** METTL3, SIRT1, Endometriosis, m6A, Epigenetic modification, Cellular senescence

## Abstract

**Background:**

Endometriosis (EMs), the ectopic planting of functional endometrium outside of the uterus, is a leading cause of infertility and pelvic pain. As a fundamental mRNA modification, N6-methyladenosine (m6A) participates in various pathological processes. However, the role of m6A RNA modification in endometriosis remains unclear. The present study explores METTL3-mediated m6A modification and the mechanisms involved in endometriosis.

**Methods:**

The dominant m6A regulators in EMs were analysed using RT‒PCR. Candidate targets and possible mechanisms of METTL3 were assessed by m6A-mRNA epitranscriptomic microarray and RNA sequencing. A primary ESCs model was employed to verify the effect of METTL3 on m6A modification of SIRT1 mRNA, and the mechanism was elucidated by RT‒PCR, Western blotting, MeRIP, and RIP assays. CCK-8 viability assays, Transwell invasion assays, EdU proliferation assays, wound healing migration assays, and senescence-associated β-galactosidase staining were performed to illuminate the potential biological mechanism of METTL3 and SIRT1 in ESCs in vitro. An in vivo PgrCre/ + METTL3 −/− female homozygous mouse model and a nude mouse xenograft model were employed to further investigate the physiologic consequences of METTL3-mediated m6A alteration on EMs.

**Results:**

Our data show that decreased METTL3 expression significantly downregulates m6A RNA methylation levels in ESCs. Silencing m6A modifications mediated by METTL3 accelerates ESCs viability, proliferation, migration, and invasion in vitro. The m6A reader protein YTHDF2 binds to m6A modifications to induce the degradation of SIRT1 mRNA. SIRT1/FOXO3a signalling pathway activation is subsequently inhibited, promoting the cellular senescence of ESCs and inhibiting the ectopic implantation of ESCs in vitro and in vivo.

**Conclusions:**

Our findings demonstrate that METTL3-mediated m6A methylation epigenetically regulates the ectopic implantation of ESCs, resulting in the progression of endometriosis. Our study establishes METTL3-YTHDF2-SIRT1/FOXO3a as a critical axis and potential mechanism in endometriosis.

**Supplementary Information:**

The online version contains supplementary material available at 10.1186/s12967-023-04209-0.

## Introduction

Endometriosis (EMs) is characterized by the presence of ectopic endometrial glands and stroma with implantation function is observed outside the uterine cavity [1]. Although EMs is a benign disease, complications, such as abnormal ovulation and pelvic adhesion, may cause infertility. However, twenty percent of women of childbearing age worldwide are at risk for physical and mental health complications due to menorrhagia and prolonged dysmenorrhea [2, 3]. Ectopic endometrium exhibits different features compared with normal endometrium, such as increased ability of invasion, adhesion, and release of cytokines [4, 5]. Therefore, it is important to explore mechanistic differences in normal endometrium compared with ectopic endometrium in EMs.

A prominent RNA modification termed N6-methyladenosine (m6A) is associated with several common cancers and diseases due to its aberrant expression [6]. The METTL3-METTL14 complex, VIRMA, RBM15, and ZC3H13 are all “m6A writers” [7] that comprise the methyltransferase complex that catalyses the conversion of m6A, METTL3 is the central catalytic core of the complex. “m6A erasers”, such as FTO and ALKBH5, facilitate the reversible removal of the “m6A writer” complex components [8]. In addition, several proteins called “m6A readers”, including the YTH domain family and the HNRNP or IGF2BP protein family, directly bind to m6A and mediate its function [9]. Studies have linked m6A and its associated regulatory proteins to various malignancies [10]. Recent studies have reported that m6A-modified mRNAs regulate the progression of EMs. For example, METTL3-mediated m6A modification prevents the formation of EMs by restricting the maturation of pri-miR126 [11]. In addition, glycolysis and metastasis mediated by the FTO/ATG5/PKM2 axis regulate the development of EMs [12]. However, the underlying mechanism of the complete m6A modification process in EMs still needs to be completely elucidated.

Cellular senescence, or cessation of the cell cycle, is commonly reported to both promote and inhibit functions in human diseases [13], and evidence suggests that cellular damage is a common trigger for the modulation of the expression of genes involved in cellular senescence. DNA damage [14], telomere shortening [15], and epigenetics [16] are leading hallmarks of cellular senescence caused by cellular damage. Activation of the canonical p53-p21 pathway [17] is the primary source of senescent cellular stress, which manifests morphologically as increased cell size with increased stress granules. Fibroblast senescence can also be modulated by increased Lamin b1 expression levels and the promotion of fibrotic and inflammatory phenotypes [18]. Senescence escape can result in deleterious biological consequences, including the formation of malignant tumours, chronic inflammatory diseases, and immune deficiency disorders. However, limited research has focused on the relationship between EMs and cellular senescence, and this topic should be further explored.

In this study, we found that prevention of METTL3-mediated m6A modification promotes the progression of EMs. By regulating m6A modifications in SIRT1 mRNA, METTL3 alters SIRT1 mRNA stability through YTHDF2 recognition, thereby promoting cellular senescence through the SIRT1/FOXO3a signalling pathway to inhibit EM progression in vitro and in vivo. Our study describes the mechanism of m6A modification in endometrial stromal cells and highlights a potential clinical marker of endometriosis.

## Materials and methods

### Patients and samples

Thirty cases of fresh ovarian ectopic endometrial tissues (EC) and 30 matched cases of eutopic endometrial specimens (EU) were collected from patients with ovarian endometriosis. Thirty samples of normal control endometrial tissues (NC) were collected from patients with cervical intraepithelial neoplasia, excluding endometriosis. All specimens were collected during the proliferative phase of the menstrual cycle. Patients who did not receive hormonal treatments for approximately three months underwent surgery at the Second Affiliate Hospital of Harbin Medical University with confirmation by postoperative pathology. Written informed consent was obtained from all patients. This research was administrated and approved by the Second Affiliate Hospital of Harbin Medical University (Ethics number: KY2022-115).

### RNA m6A quantitative assay

Relative m6A levels among total RNAs were evaluated using a m6A RNA Methylation Quantification Kit (Colorimetric) (P-9005, Epigentek, USA). Total RNA was extracted using TRIzol extraction reagent (Ambion, USA), strips were treated with 200 µg/μl total RNA. According to the instructions, RNA was allowed to bind to the strip upon incubation in high binding solution at 37 °C for 90 min. As reported in a previous study, capture and detection antibodies were added to detect the m6A colorimetric levels by reading the absorbance at a wavelength of 450 nm [19]. The relative absorbance was quantified for five experimental replicates for each reaction.

### m6A dot blot assays

m6A dot blotting was performed as previously described [20]. Total RNA was harvested as previously described with RNA quantity monitoring. RNA was denatured by heating at 95 °C for 10 min and then preserved on ice. Then, mRNAs were blotted onto an N^+^ nylon membrane (FFN10, Beyotime, China). Afterwards, the membrane was treated with ultraviolet cross-linking and blocked with 5% nonfat milk in PBST for one hour at room temperature. The membranes were incubated with a m6A-specific antibody (1:2000, 202003, Synaptic Systems) for reactivation at 4 °C overnight. After washing, the membranes were incubated with a goat anti-rabbit IgG HRP antibody (1:5000, bs-40295G-HRP, Bioss) with gentle shaking for 1 h at room temperature. After washing, the membranes were incubated with an ECL detection reagent (MA0186, Meilunbio, China) and visualized using the detection system.

### RNA extraction, reverse transcription, and real-time PCR

Total RNA was extracted from all samples using TRIzol reagent (Ambion, USA) and reverse transcribed to cDNA by a PrimeScript™ RT Reagent Kit with gDNA Eraser (RR047A, Takara, Japan). RT‒qPCR was performed using TB Green^®^ Premix Ex Taq™ (RR820A, Takara, Japan). The RT‒qPCR program was as follows: 40 cycles at 37 °C for 15 min, 60 °C for 5 s, and 72 °C for 30 s. All relative mRNA expression levels were analysed using the 2−ΔΔCt method. All experiments were performed according to the instructions with three repeats.

The primers used are listed in Additional file 1: Table S1.

### Western blotting

After rinsing in PBS, all samples were treated with RIPA buffer (R0010, Solarbio, China) for complete lysis. Then, 1% PMSF (P0100, Solarbio, China) was added, and samples were incubated on ice for 30 min. After centrifugation, we measured the protein concentration with a BCA protein assay kit (MA0082, Meilunbio, China). Protein was separated by SDS‒PAGE (P0015L, Beyotime, China) and transferred to PVDF membranes (Roche, USA). After blocking in quick block buffer for 30 min, the membranes were incubated with primary antibody. Samples were incubated in secondary goat anti-rabbit IgG HRP antibodies (1:5000, bs-40295G-HRP, Bioss) for one h at room temperature. An ECL detection reagent (MA0186, Meilunbio, China) was added to the blots, and imaging was performed on a ChemiDocMP (Bio-Rad) imager. The membranes were incubated with the following primary antibodies at 4 °C overnight: anti-METTL3 (1:2000, ab195352, Abcam), anti-YTHDF2 (1:5000, 24744-1-AP, Proteintech), anti-p53 (1:5000, 10442-1-AP, Proteintech), anti-p21 (1:1000, 10355-1-AP, Proteintech), anti-Lamin b1 (1:2000, 12987-1-AP, Proteintech), anti-FOXO3a (1:2000, 28755-1-AP, Proteintech), anti-SIRT1 (1:2000, 13161-1-AP, Proteintech) and GAPDH (1:5000, 10494-1-AP, Proteintech).

### Isolation and culture of primary endometrial stromal cells

Endometrial stromal cells were isolated from ectopic, eutopic, and normal control endometrial tissues and were named ESCs, EuSCs, and NESCs, respectively, as described in a previous study [21]. Samples were washed twice with phosphate-buffered saline (PBS), minced, and coated with 1 mg/ml collagenase type IV (BS165, Biosharp, The Netherlands). Samples were incubated at 37 °C with mild shaking for two hours in a culture flask. Next, a 40-mm sieve was used to remove debris and other cells, such as endometrial epithelial cells, and the solution that passed through was centrifuged for 10 min at 1000 r/min. Cell precipitates were collected by removing the supernatant. The cells were grown in a 5% CO_2_ humidified atmosphere at 37 °C in DMEM/F12 (MA0214, Meilunbio, China) supplemented with 10% foetal bovine serum (BI, Israel).

### Cell transfection for gene silence and overexpress

Small interfering RNAs (siRNA) explicitly targeting METTL3 (si-METTL3), SIRT1 (si-SIRT1), YTHDF1 (si-YTHDF1), YTHDF2 (si-YTHDF2), YTHDF3 (si-YTHDF3) and negative control (si-NC) were obtained from Hanbio (Shanghai, China) and used as previously described [22]. The METTL3 (oe-METTL3), YTHDF2 (oe-YTHDF2), and negative cintriol (oe-NC) were constructed and designed on the overexpression plasmid and synthesized by Hanbio (Shanghai, China). The siRNAs were transfected into the cells using RNA Fit (Hanbio, Shanghai, China) according to the protocol, and plasmid transfection was achieved using LipoFiter^TM^3.0 Liposomal Transfection Reagent (Hanbio, Shanghai, China) as previously described [22]. siRNA sequences are listed in Additional file 1: Table S2.

### RNA-seq

Wild-type ESCs and ESCs transfected with the oe-METTL3 plasmid were collected and rinsed twice with PBS. RNA extraction was performed as previously described, and RNA sequencing was performed by Allwegene Tech (Beijing, China). Illumina next generation sequencing was used as the high-throughput sequencing platform, and HiSeq/NovaSeq PE150 was employed as a sequencing strategy. A total of 148875607 raw read pairs were measured after quality control, resulting in 146641251 clean read pairs. DESeq2 was used to calculate the transcripts with an adjusted *p* value ≤ 0.01, and genes with a fold change ≥ 1 were recognized as targets.

### m6A epitranscriptomic microarray array analysis

Wild-type ESCs and ESCs transfected with the oe-METTL3 plasmid were analysed by Aksomics Company (Shanghai, China) using a human m6A epitranscriptomic microarray and mRNA microarray at Arraystar m6A single-base resolution [23]. Total RNA was treated with anti-N6-methyadenosine (m6A) for immunoprecipitation. After merging immunoprecipitated magnetic beads and recovered supernatant, Arraystar RNA was used as labelled RNA and hybridized with Arraystar Human mRNA & lncRNA Epitranscriptomic Microarray (8 × 60 K, Arraystar). Arrays were scanned with Agilent Scanner G2505C in the two-colour channel, and credit analysis was performed. Genes with |FC|≥ 2 and *p* value < 0.05 were chosen as differentially m6A-methylated target RNAs.

### Cell viability assay

One hundred microlitres of culture medium containing 3 × 10^4^ cells was cultured in 96-well plates for 24 h. Ten microlitres of Cell Counting Kit-8 (CCK-8) reagent (MA0218-2, Meilunbio, China) was added to each well. After incubating for an additional two hours in a humidified atmosphere at 37 °C with 5% CO_2_, cellular absorbance at 450 nm was assessed using a spectrophotometer. Each reaction was repeated thrice.

### Cell proliferation assay

Briefly, 2 × 10^5^ ESCs from each well were cultured in a 24-well plate. One microlitre of EdU reagent (MA0425, Meilunbio, China) was added to 1 ml of culture medium. After incubation in a humidified atmosphere at 37 °C for three hours, the cells were immobilized with 4% PFA, permeabilized with 0.3% Triton X-100 in PBS, and stained with the click reaction solution, which is comprised of 555 azide and Hoechst 33342 stain. The results were imaged by fluorescence microscopy. Specifically, red fluorescence was detected at 555/567 nm, and blue fluorescence was detected at 346/460 nm. Three images were randomly obtained for each reaction.

### Cell migration assay

A total of 1 × 10^6^ cells from each well were cultured in a 6-well plate until 80–90% confluent. Scratches were created using a sterile 200-μl pipette tip in a straight and cautious manner. Photographs were taken at 0 and 24 h, and three replicates were performed for each experimental condition.

### Cell invasion assay

Transwell chambers that were precoated with Matrigel (BD Biosciences, USA) were used to separate ESCs. Specifically, the upper section contained DMEM/F12 with 0.1% FBS, and the bottom chamber contained DMEM/F12 with 20% FBS. After incubation in a humidified atmosphere at 37 °C and 5% CO_2_ for 24 h, cells that did not invade were removed. Cells invading the surface of the bottom chamber were fixed and stained with 0.1% crystal violet (G1062, Solarbio, China). After air drying, the invading cells were photographed using a microscope and quantified by counting five random fields.

### Senescence associate β-galactosidase staining

SA-β-Gal staining was performed as previously described [24] using a Senescence β-Galactosidase Staining Kit (C0602, Beyotime, China). Briefly, cultured cells were rinsed twice with PBS and fixed at room temperature for 15 min in β-galactosidase staining fixative. Fixed cells were stained with SA-β-Gal staining solution A, B, C, and X-gal as per the instructions at 37 °C overnight, and images were captured using a microscope camera. Three experimental replicates were performed.

### RNA immunoprecipitation-PCR (RIP-qPCR) assay

The RNA Immunoprecipitation Kit (P0102, Geneseed, China) was used to conduct the RIP experiment according to the manufacturer’s protocol [25]. ESCs were lysed in an IP lysis buffer, and the resulting cell lysate was separated into anti-METTL3, anti-YTHDF2, anti-IgG, and input samples. Protein A/G beads coated with identical amounts (5 g) of specific antibodies were applied to incubate cell lysates. After washing, the lysates were digested with protease and RNase inhibitors for purification. RT‒qPCR was performed to measure the target RNA levels.

### Methylated RNA immunoprecipitation-PCR (MeRIP-qPCR) analysis

RNA isolation was performed as previously described. A riboMeRIP m6A Transcriptome Profiling Kit (C11051-1, Ribobio, China) was used for the MeRIP experiment as previously described [26]. Briefly, total RNAs were sheared into approximately 100- to 150-nucleotide fragments using the RNA Fragmentation Buffer at 94 °C for 3 min; we preserved a small amount and marked them as the input RNAs. Prewashed magnetic beads A/G were precoated with m6A-specific antibody for 30 min at room temperature. Then, the beads were mixed with the fragmented RNA for immunoprecipitation. Relative gene expression was determined by qRT‒PCR as previously described.

### mRNA stability assessment

After transfection, ESCs were treated with 5 μg/ml actinomycin D (M4881, Abmole, USA) for different time periods: 0, 3.0 h, and 6.0 h. Then, the cells were collected for RNA extraction, and RT‒PCR analysis was performed as previously described [27].

### Immunohistochemistry and immunofluorescent staining

Immunohistochemistry was performed as previously described [28]. Briefly, human and mouse sections were dewaxed and rehydrated followed by antigen retrieval using Tris antigen-retrieval buffer. Then, the sections were incubated with primary and secondary antibodies. Immunofluorescence staining was performed as previously described. After incubation with primary antibodies, the cultures were incubated with Alexa Fluor 488 (1:200, ab150077, Abcam) or Cy3 (1:500, ab6939, Abcam) conjugated secondary antibodies, and images were captured with experiments repeated thrice.

### Endometriosis CKO-mouse donor-receiptor grafting model

For all animal studies, animals were randomly distributed and showed no size or appearance differences at the onset of the experiments. A Cre/Lox system was used to generate a tissue-specific knockout model. Endometriosis CKO mouse models were constructed as previously described [29, 30]. PRcre mice (C001035, Cyagen, China) were crossed with METTL3fl/fl homozygous mice (TOS171205 WZ1, Cyagen, China) to generate PRCre/ + METTL3 −/− biogenic mice in which METTL3 expression is abrogated in PR-expressing cells. Mice were genotyped to confirm METTL3fl/fl homozygosity together with the PgrCre/ + transgene using PgrCre/ + -specific primers (F: 5′-GCGCTAAGGATGACTCTGGTC-3′ and R: 5′-CCCTTCTCATGGAGATCT GTC-3′) and METTL3fl/fl-specific primers (F: 5′-TCCAAGAGTCTAATATCC ACCAGCAC-3′ and R: 5′-TGATCAGCAAATGATGGTCCCAG-3′). Mice were randomly divided into donor and recipient groups in both the PRCre/ + METTL3 −/− group (CKO-METTL3 group) and METTL3f/f group (Control group). Donor mice were injected with oestradiol (E2) (1 μg/ml) every three days at six weeks of age for two weeks. After 2 weeks, the uterine horns of the donor mice were surgically removed and dissected in saline to completely expose the endometrial surface, transplanted to the intraperitoneal cavity of the recipient mice to generate endometriosis. Mice were sacrificed on Days 3, 5, and 7 after receiving an intraperitoneal injection, and endometrial lesions were recorded through observation. Lesion volumes were computed using the prolate ellipsoid geometric model: (length × width)^2^/2. All animal experiments were approved by the Ethics Committee of the Second Affiliate Hospital of Harbin Medical University (ethics number: SYDW2022-080).

### Nude mice xenografts model

Female BALB/c nude mice purchased from the Charles River Laboratory were maintained in SPF conditions for one week before use. The experiments were approved by the Ethics Committee of the Second Affiliate Hospital of Harbin Medical University. For in vivo studies, the shMETTL3 sequence was subcloned into adeno-associated virus (AAV) construct 9 (AAV9). Recombinant AAV9 was manufactured by GenePharma (Shanghai, China). The ectopic endometrium was obtained from a 28-year-old woman who had undergone an operation for ovarian endometriosis. The tissue sample was washed twice with PBS and cut into three 3- to 5-mm pieces, and suspended by PBS. Briefly, 200 μl of suspension was injected under the bilateral axilla in each mouse as previously described [31]. The animals were administered intraperitoneal injections of 30 mg/kg 17-oestradiol every three days after the endometrial injections. Xenografts were generated under bilateral axillae after 14 days with tumour volumes of approximately 10 mm^3^.

Subsequently, nude mice with lesions were randomly divided into two groups. In the study, seven mice were administered intratumor injections of METTL3 shRNA AAV, whereas the other seven mice received control shRNA AAV. Mice were given injections with 5 μL of AAV daily for 10 days [32]. Every five days, the tumour was measured using a slide calliper, and the volume was determined using the method previously described. Twenty days after virus injection, all mice were anaesthetized and euthanized by cervical dislocation, and tumours were removed and measured.

### Statistical analysis

All the data were analysed as the mean ± SD, and two-tailed Student’s t test and one-way ANOVA by SPSS (v.16.0) were performed using GraphPad software (v.9.0.0) to determine statistical significance. All experiments were performed at least thrice. Here, ns indicates not significant (*p* ≥ 0.5); *p < 0.05 denotes a moderate statistically significant result; **p < 0.01 denotes a statistically significant result; and ***p < 0.001 indicates a highly statistically significant result.

## Results

### Endometriosis leads to decreased m6A modification levels and METTL3 serves as a significant regulator

**Fig. 1:**
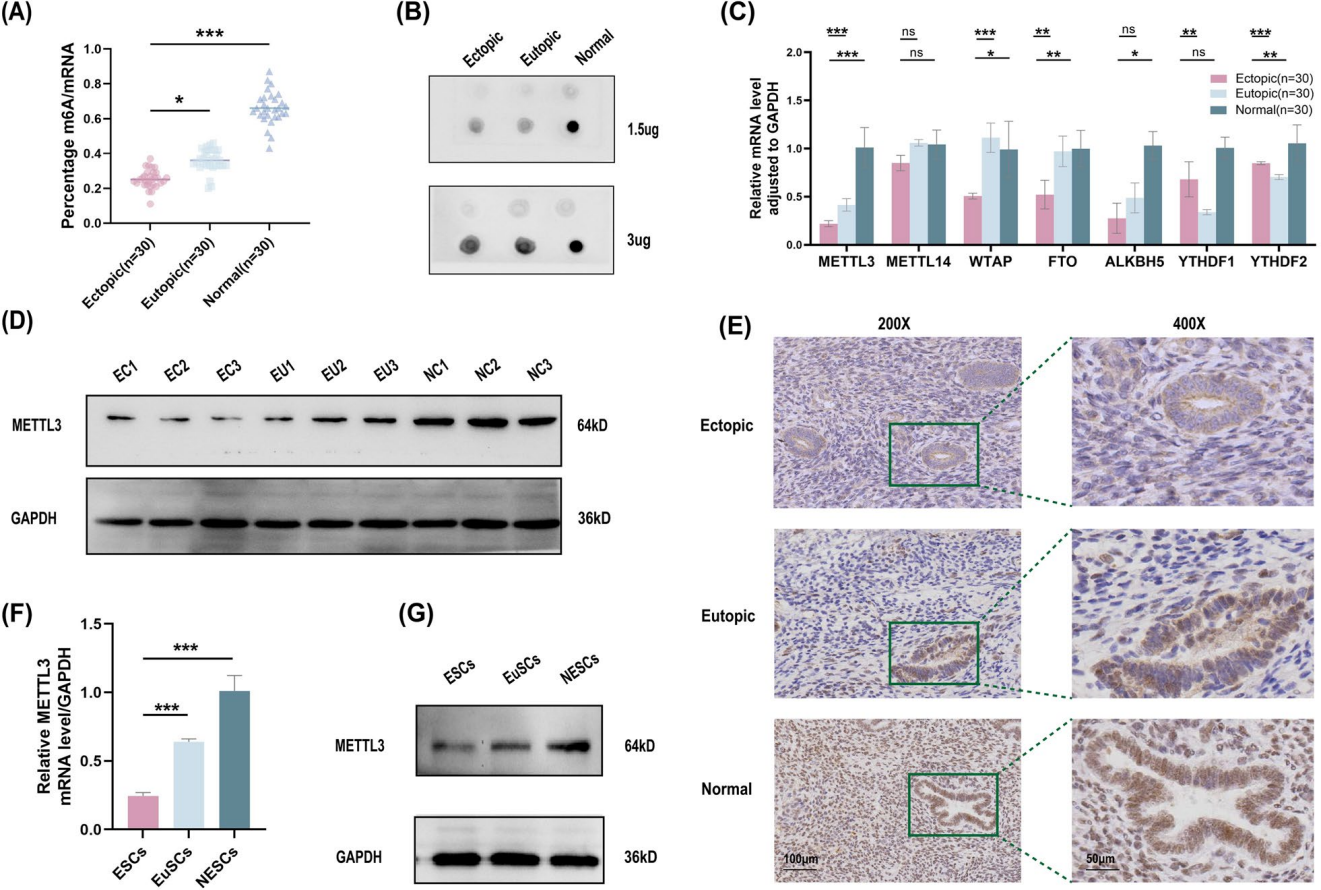
METTL3 shows decreased expression in endometriosis specimens and primary ESCs. **A** The mRNA m6A levels in human ectopic with strict match eutopic endometium, and normal control endometrium of 30 paired specimens were detected by ELISA via an m6A RNA methylation colorimetric quantification kit. **B** The global m6A level in mRNA in human ectopic, eutopic, and normal control endometrium measured by m6A dot blot assay with anti-m6A antibody. **C** The mRNA level adjusted to GAPDH of methyltransferases, demethylases and N6-methyladenosine readers in EMs and paired tissues by RT-qPCR. **D** Protein level of METTL3 in EMs and paired specimens by western blotting, using GAPDH as an internal control. **E** Representative images illuminate METTL3 expression in EMs and paired tissues by immunohistochemistry (IHC) (scale bars = 100 μm and 50 μm). Relative expression of METTL3 in primary Ectopic(ESCs), Eutopic(EuSCs), and Normal control(NESCs) endometrium cells in mRNA level by RT-qPCR **(F)** and protein level by western blotting **(G)**, using GAPDH as an internal control. All above result were shown in means ± SD, ns, *p* ≥ 0.05; *p < 0.05; **p < 0.01; ***p < 0.001

To elucidate the relevance of m6A modification in EMs, the degree of m6A modification was evaluated in samples from the ectopic (EC) group (n = 30), eutopic (EU) group (n = 30), and normal control (NM) group (n = 30). The results indicated that m6A modification levels were decreased in EC samples compared to EU and NM endometrium samples (Fig. 1A, B). Additionally, we detected familiar m6A regulators, including writers (METTL3, METTL14, and WTAP), erasers (FTO and ALKBH5), and readers (YTHDF1 and YTHDF2), in EC, EU, and NM endometrial samples. RT‒qPCR analysis results showed that METTL3 mRNA levels were the most significantly reduced in the EC group. However, no statistically significant alterations in METTL14, YTHDF1, and ALKBH5 were noted in the ectopic group compared with the eutopic or normal group (Fig. 1C). Additionally, METTL3 protein levels were significantly decreased in EC samples compared to the EU and NM groups as assessed by western blotting assay (Fig. 1D). To properly explore METTL3 activation in EMs, we performed immunohistochemistry (IHC) staining to examine METTL3 expression in ectopic, eutopic, and normal endometrial specimens (Fig. 1E). We found the same expression trend among the three groups in our study. We subsequently investigated whether METTL3 expression correlated with the clinicopathological characteristics of EMs. METTL3 expression was independent of dysmenorrhea and age. Endometriosis patients at stages III + IV (according to the revised American Fertility Society classification, r-AFS) or without deeply infiltrating endometriosis and cyst size ≥ 3 cm in diameter showed reduced METTL3 expression (Table 1). Furthermore, we assessed METTL3 expression in the following primary cell groups: ESCs (ectopic endometrial stromal cells), EuSCs (eutopic endometrial stromal cells), and NESCs (normal control endometrial stromal cells). METTL3 mRNA and protein levels were reduced in ESCs compared with EuSCs and NESCs as demonstrated qPCR and western blotting analyses (Fig. 1F, G). Taken together, these studies revealed that METTL3 expression levels and m6A RNA modifications were reduced in EMs.

**Table 1:** Clinical characteristics and METTL3 expression level in Endometriosis

Clinicopathological Features	Cases (n)	METTL3 relative expression	*p* value
r-AFS stage			
I, II	13	2.50 ± 0.33	< 0.001***
III, IV	17	1.03 ± 0.15	
Cyst size (cm)			
< 3	14	2.05 ± 0.26	< 0.001***
≥ 3	16	1.33 ± 0.14	
DIE			
No	21	1.55 ± 0.33	< 0.01**
Yes	9	1.94 ± 0.30	
Dysmenorrhea			
No symptoms	10	1.74 ± 0.18	0.216
Moderate	13	1.65 ± 0.17	
Severe	7	1.59 ± 0.14	
Age			
≤ 25	10	1.63 ± 0.19	0.784
25–35	14	1.69 ± 0.14	
35–45	6	1.67 ± 0.17	

### METTL3 inhibits the biological progression of EMs by enhancing cellular senescence in vitro and in vivo

**Fig. 2:**
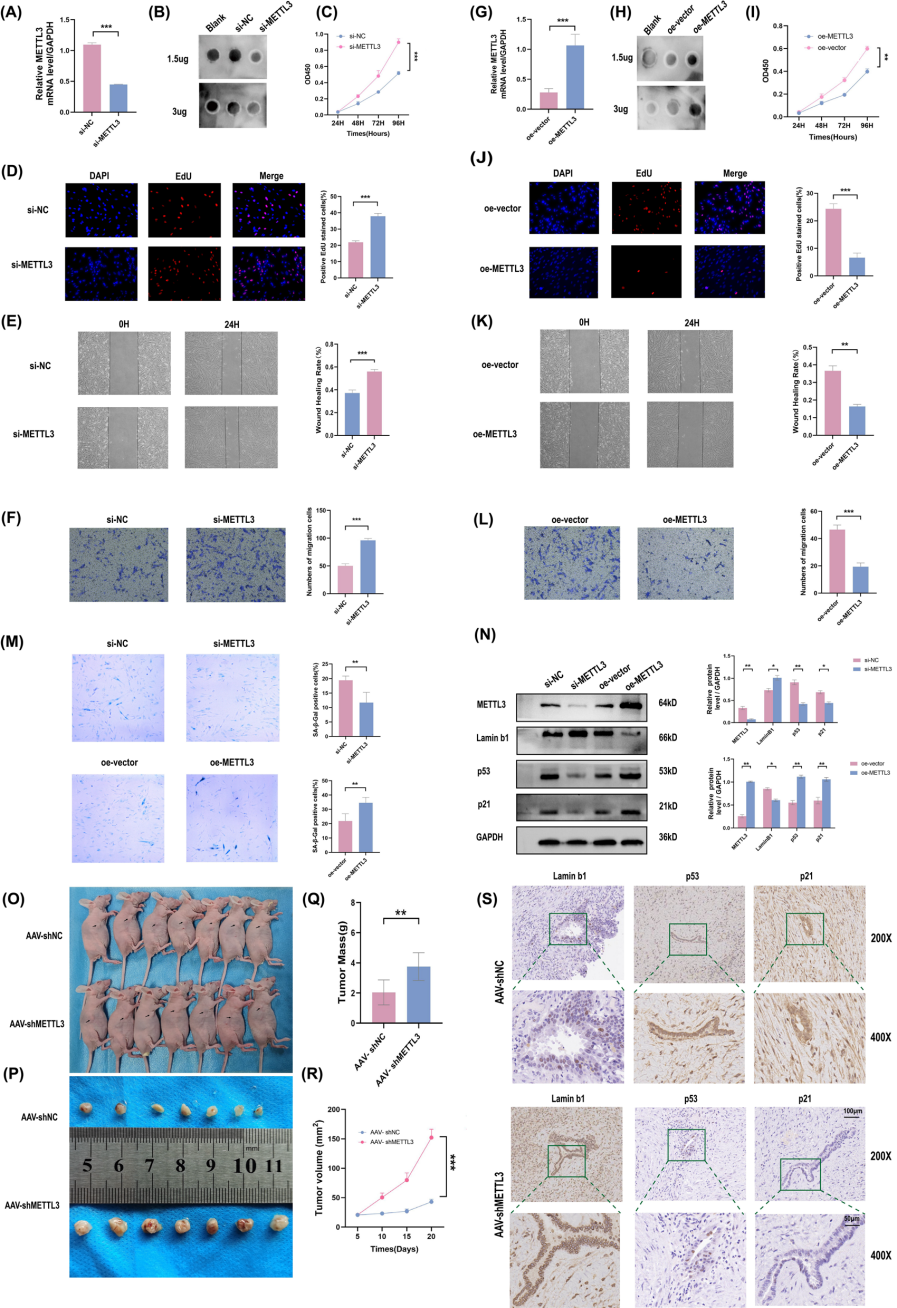
METTL3 mediate m6A methylation inhibits the progression of EMs by promoting cellular senescence in vitro and in vivo. **A** The measurement of the knockdown efficiency of si-METTL3 transfected ESCs in mRNA level by RT-qPCR. **B** The global m6A levels in si-METTL3 transfected ESCs detected by m6A dot blot assay with anti-m6A antibody. The increasing ability of viability, proliferation, invasion, and migration of ESCs with si-METTL3 transfection was detected by CCK8 **C**, EdU staining **D**), wound healing **E** and Transwell **F** assay. **G** The mRNA level of METTL3 in oe-METTL3 transfected ESCs by RT-qPCR. **H** The mRNA N6-methyladenosine level in oe-METTL3 transfected ESCs by m6A dot blot. **I–L** CCK8, EdU, wound healing, and Transwell assay showed the decreasing ability of viability, proliferation, invasion, and migration of ESCs with oe-METTL3 transfection in reverse. **M** Level of cellular senescence in ESCs transfect with si-NC, si-METTL3, oe-vector and oe-METTL3 by SA-β -Gal staining. **N** The protein level of METTL3 and senescence biomarkers in ESCs transfect with si-NC, si-METTL3, oe-vector, and oe-METTL3 by western blotting, using GAPDH as an internal control. **O** Mass morphology of AAV-shNC and AAV-shMETTL3 mice. **P** The xenografts were isolated and measured by caliper in AAV-shNC and AAV-shMETTL3 mice. Xenografts’ weight **Q** and volume **R** were measured and analyzed. **S** Representative staining images for cellular senescence biomarkers (Lamin b1, p53, and p21) in AAV-shNC(up) and AAV-shMETTL3 group(down) by IHC. (Scale bars = 100 μm and 50 μm). All above result were shown in means ± SD, ns, p ≥ 0.05; *p < 0.05; **p < 0.01; ***p < 0.001

Based on the above findings, METTL3 was downregulated in EMs samples and ESCs. We hypothesized that METTL3 serves as a disease inhibitor in EMs. To explore whether METTL3 regulates RNA m6A modification in ESCs, si-METTL3 transfection efficiency was measured, as shown in Fig. 2A. Then, total RNA m6A modification levels were measured, and the results demonstrate that METTL3 deletion decreased m6A modification levels (Fig. 2B). To determine the function of METTL3 in EMs, METTL3 was knocked down and overexpressed in ESCs. The role of METTL3 in cell viability, invasion, proliferation, migration as well as and the level of cellular senescence was assessed. As expected, METTL3 depletion markedly enhanced the viability, proliferation, and migration ability of ESCs (Fig. 2C–F). In addition, METTL3 expression increased the modification level (Fig. 2G, H) and significantly decreased the viability, proliferation, and migration ability of ESCs (Fig. 2I–L). The results of SA-β-gal staining showed that METTL3 knock down effectively rescued METTL3-induced promotion of cellular senescence in ESCs. The senescent cells positivity rate was significantly augmented and the cell size was enlarged after METTL3 overexpression (Fig. 2M). To further detect the effect of METTL3 expression on ESCs senescence, p53, p21, and Lamin b1 were chosen as markers in our research (Fig. 2N). The expression of the p53 and p21 proteins was significantly enhanced with METTL3 overexpression, while the expression of Lamin b1 protein decreased, indicating a trend towards cellular senescence. In contrast, ESCs with METTL3 knocked down showed the opposite trend.

To confirm that METTL3 drives the formation of EMs in vivo, nude mouse models with subcutaneous xenografts were employed. In these experiments, Day 0 was two weeks after the establishment of subcutaneous xenografts.

After AAV injection for five days, we recorded the volumes of the xenografts every 5 days (Days 5, 10, 15, and 20). As shown, AAV-shMETTL3 intervention robustly enhanced the expansion of EMs xenografts in nude mice (Fig. 2O, P). All EMs xenografts were delicately extracted and weighed on “Day 20”. Xenografts from the AAV-shMETTL3 group were significantly larger and heavier than those from the AAV-shNC group (Fig. 2Q, R). Whether AAV-shMETTL3 caused a similar cellular senescence signalling shift in vivo was investigated. As shown by immunohistochemistry, a dramatic increase in the cellular senescence inhibitor (Lamin b1) and an decrease in cellular senescence promoters (p53 and p21) were observed in EMs xenograft tissues injected with AAV-shMETTL3 (Fig. 2S).

### Analysis of downstream targets and functions of METTL3 in EMs

**Fig. 3:**
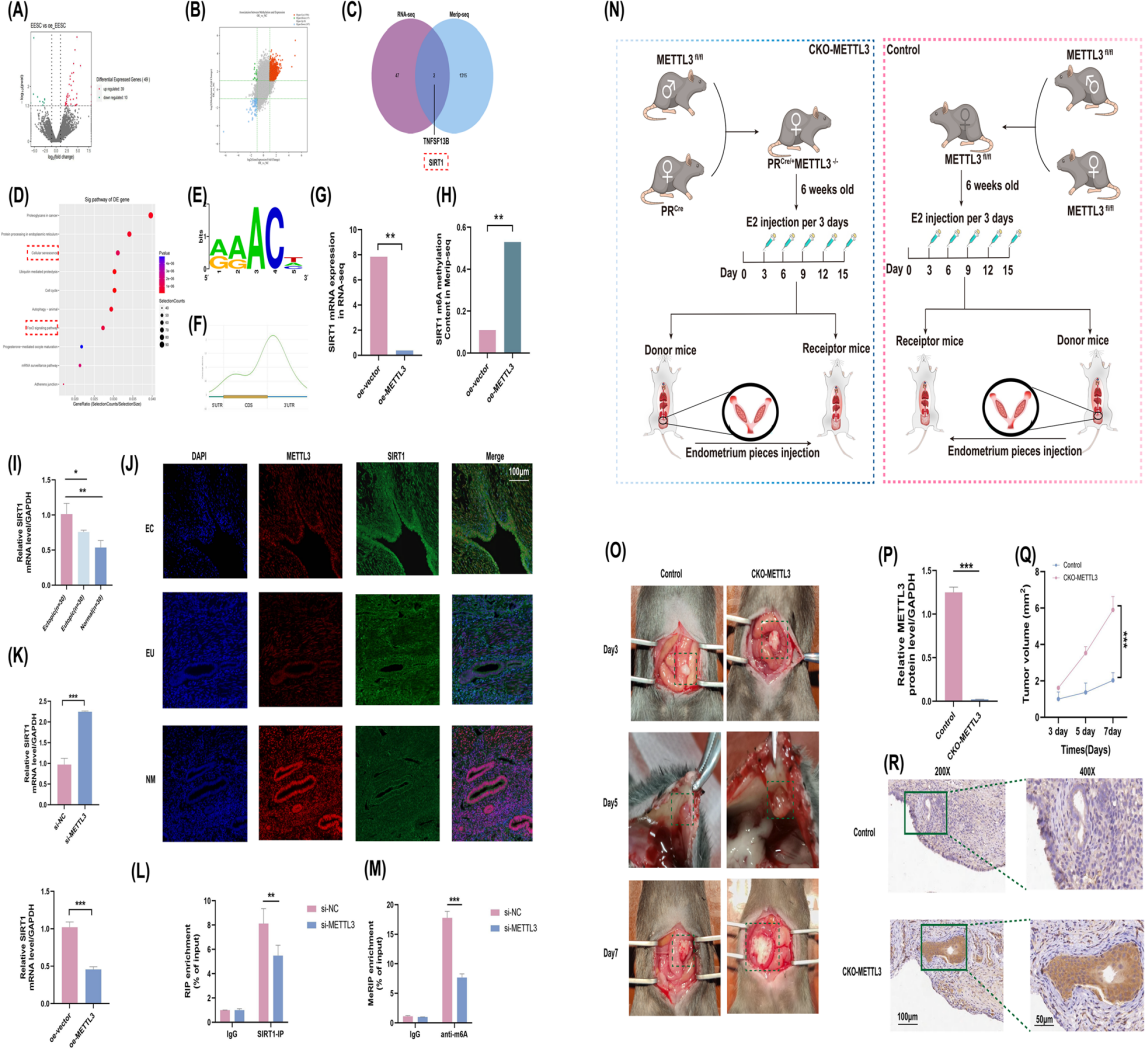
SIRT1 as a downstream target and negatively regulated by METTL3-mediated m6A modification in vitro and in vivo. **A** Volcano plots for differentially expressed mRNAs between matched oe-vector and oe-METTL3 transfected ESCs by RNA-seq. (|Fold Change|≥ 1, *p* < 0.001, Red point: up-regulated mRNAs, blue point: down-regulated mRNAs, the grey point indicated not differential expressed). **B** Volcano plots for differential methylated m6A modification transcripts between matched oe-vector and oe-METTL3 transfected ESCs by m6A epitranscriptomic microarray.(|Fold Change|≥ 1). Hyper-Up, up-regulated m6A modified mRNAs of |Fold Change|≥ 2; Hyper-Down, down-regulated m6A modified mRNAs of |Fold Change|≥ 2; Hypo-Up, up-regulated m6A modified mRNAs of |Fold Change|< 2; Hypo-Down, down-regulated m6A modified mRNAs of |Fold Change|< 2. **C** Venn showed SIRT1 at the intersection of RNA-seq and m6A epitranscriptomic microarray. **D** Top results of KEGG enrichment analysis. KEGG, Kyoto Encyclopedia of Genes and Genomes. **E** The m6A motif of ESCs identifified by m6A epitranscriptomic microarray. **F** m6A peak distribution of mRNA in m6A epitranscriptomic microarray. **G** The decreasing SIRT1 expression level of oe-METTL3 transfected ESCs in RNA-seq. **H** The increasing m6A mythylation level of oe-METTL3 transfected ESCs in m6A epitranscriptomic microarray. **I** The mRNA level of SIRT1 in EMs and paired tissues by RT-qPCR. **J** Representative IF staining of METTL3 and SIRT1 expression in Ectopic, Eutopic, and normal endometrium tissues. (Red fluorescence: METTL3; Green fluorescence: SIRT1; Blue fluorescence: DAPI; scale bar: 100 μm). **K** mRNA level of SIRT1 in METTL3 knockdown(up) and overexpression(down) by RT-qPCR. **L** Reduction enrichment level of SIRT1 in ESCs after METTL3 silencing by RIP-qPCR. **M** Decreasing m6A modification level of SIRT1 transcripts during METTL3 knockdown by Merip-qPCR assay. **N** Workflow of Endometriosis CKO mice model. **O** Progression of ectopic lesions (Green rectangular range) on Days 3, 5, and 7 in control and CKO-METTL3 mice. The mRNA expression of METTL3 **P** and volume on Days 3, 5, and 7 **Q** of isolated ectopic lesions were measured and analyzed. **R** Representative IHC images for SIRT1 in Control(up) and CKO-METTL3 group(down). (scale bars = 100 μm and 50 μm). All above result were shown in means ± SD, ns, p ≥ 0.05; *p < 0.05; **p < 0.01; ***p < 0.001

To further investigate the function of METTL3 in EMs, matched oe-NC ESCs and oe-METTL3 ESCs were treated, an RNA-seq (Fig. 3A) and human m6A epitranscriptomic microarrays and mRNA microarrays (Fig. 3B) were used to explore the potentially methylated target mRNAs of METTL3. We focused on pinpointing METTL3 targets, which were identified as abnormally expressed transcripts in RNA-seq and differentially methylated transcripts in m6A epitranscriptomic microarrays. As shown in the Venn diagram, potential downstream mRNAs of METTL3 were obtained, and SIRT1 (Fig. 3C), a vital FoxO signal pathway that induces transcription factors involved in cellular senescence, autophagy, and pyroptosis processes, was identified as an important gene in this analysis. KEGG pathway analysis revealed that these differentially expressed genes were significantly enriched in the cell cycle, ubiquitin-mediated proteolysis, proteoglycans in cancer, autophagy, protein processing in the endoplasmic reticulum, FoxO signalling pathway, adherens junction, and cellular senescence (Fig. 3D). Notably, SIRT1 was highly enriched in the FoxO signalling pathway and cellular senescence, which ranked as the target enriched signalling pathways. The most prominent m6A motif, GGAC, was enriched in the m6A peaks (Fig. 3E). In particular, the m6A peaks were enriched proximal to the stop codon and 3′ untranslated region (3′ UTR) compared with peaks in the 5′ UTR and CDS region (Fig. 3F). RNA-seq data indicated SIRT1 downregulation after oe-METTL3 transfection in ESCs (Fig. 3G). m6A epitranscriptomic microarray data also showed enrichment of METTL3 methylation and the frequency of m6A after oe-METTL3 transfection in ESCs (Fig. 3H). We consider SIRT1, which was selected as a potential target, to be downstream of METTL3.

### METTL3 negatively regulates SIRT1 expression by triggering m6A modification of EMs in vitro and in vivo

To assess the regulatory roles of METTL3 and SIRT1, first, total RNA was extracted, and SIRT1 levels were determined. SIRT1 expression levels were assessed by RT‒qPCR, which showed increased expression in ectopic tissues (Fig. 3I). As shown in Fig. 3J, consistent with previous studies, ectopic endometrial lesions showed reduced METTL3 expression and increased SIRT1 expression. In contrast, normal endometrial tissues with increased METTL3 expression exhibited lower SIRT1 levels as demonstrated by immunofluorescence (IF) staining.

To confirm METTL3-mediated m6A modification of SIRT1, we assessed SIRT1 mRNA levels in METTL3-overexpressing and METTL3-knockdown ESCs. METTL3 overexpression reduced SIRT1 transcript levels in ESCs (Fig. 3K), suggesting the negative regulation of SIRT1 by METTL3. Then, we investigated the role of m6A modification in the regulation of SIRT1 by METTL3. Quantitative RIP assays indicated that SIRT1 mRNA interacts with METTL3 in ESCs. Furthermore, SIRT1 enrichment was markedly reduced in ESCs upon METTL3 knockdown (Fig. 3L). To verify that m6A modification of SIRT1 was regulated by METTL3, we performed MeRIP-qPCR experiments in ESCs with METTL3 knockdown. METTL3 knockdown attenuated the enrichment in SIRT1 levels (Fig. 3M).

To further verify that METTL3-mediated regulation of EMs was dependent on SIRT1, we constructed an endometriosis model of METTL3 deletion using a CKO mouse endometriosis model, and the entire workflow is shown in Fig. 3N. Donor mouse endometrium was grafted into the recipient peritoneal cavity (labelled Day 0). Then, we sacrificed the mice on Days 3, 5, and 7 and observed lesions in the peritoneal cavity or visceral peritoneum. The EMs lesion showed apparent enlargement in the CKO-METTL3 group compared to the control group on Days 3, 5 and 7 (Fig. 3O). Furthermore, all grafting lesions were carefully removed on Day 7. We assessed METTL3 expression by RT‒qPCR. A reduction in METTL3 expression in the grafting lesions was noted in the CKO-METTL3 group (Fig. 3P), which is consistent with the aforementioned observation (Fig. 3Q). As shown by immunohistochemistry (Fig. 3R), SIRT1 expression was dramatically increased in grafted tissues from the CKO-METTL3 group, which confirms that the negative coordination of METTL3 and SIRT1 promotes the progression of EMs.

### SIRT1 depression resists the progression of EMs caused by cellular senescence escape in METTL3-depleted ESCs

**Fig. 4:**
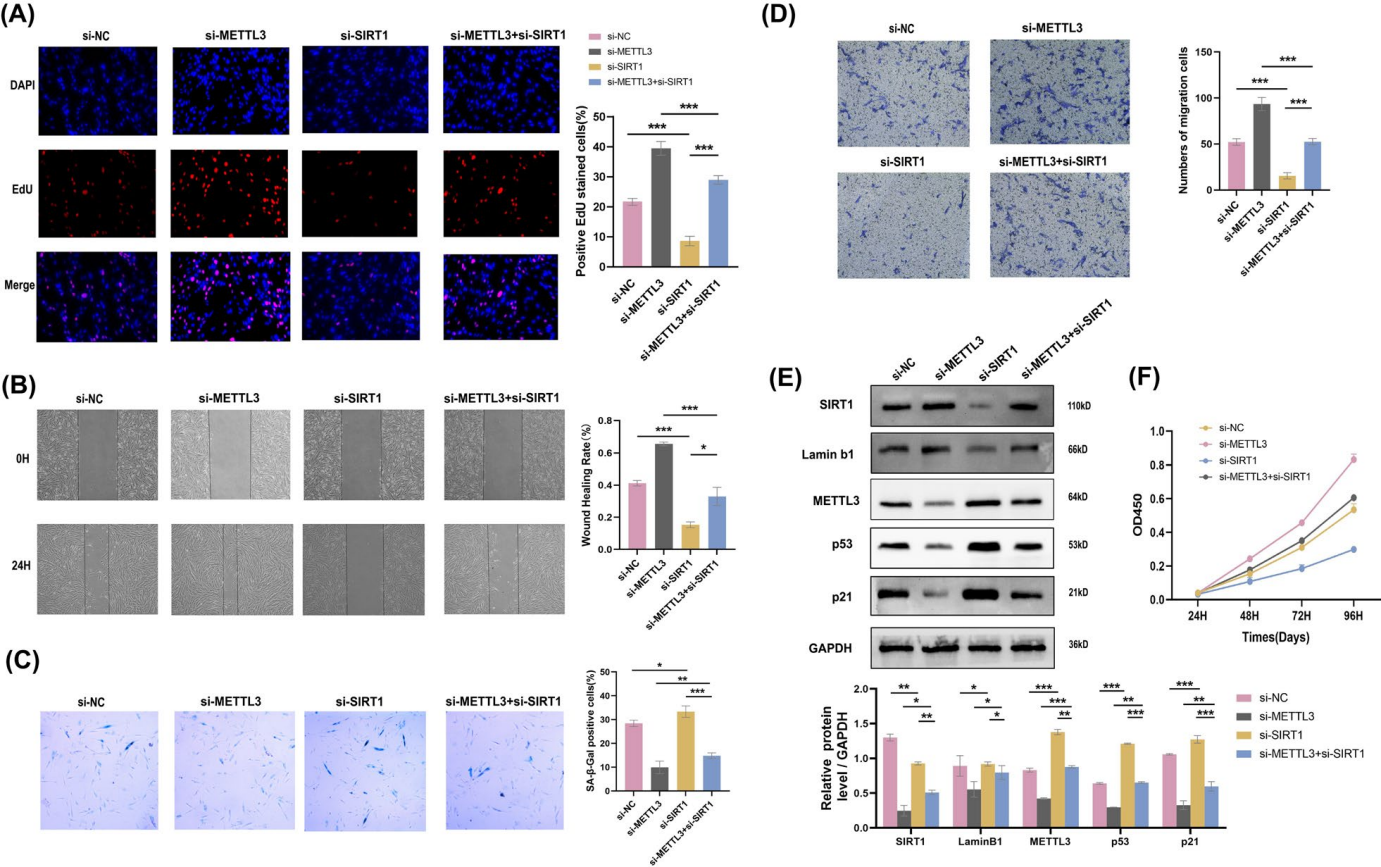
Reduction of SIRT1 resists the progression of EMs in METTL3-depleted ESCs. SIRT1 exhaustion rescued the ability of proliferation by EdU assay **A**, the ability of migration by wound healing assay **B**, the level of cellular senescence by SA-β-Gal staining **C**, the ability of invasion by Transwell assay in METTL3 depletion ESCs. **E** The protein level of METTL3, SIRT1, and senescence biomarkers in ESCs transfect with si-NC, si-METTL3, si-SIRT1, and si-METTL3 + si-SIRT1 by western blotting, using GAPDH as an internal control. **F** SIRT1 knockdown reduced the ability of viability induced by METTL3 knockdown. All above result were shown in means ± SD, ns, p ≥ 0.05; *p < 0.05; **p < 0.01; ***p < 0.001

As depicted in Fig. 4, SIRT1 knock down considerably repressed cell viability, proliferation, and migration, promoting cellular senescence as expected. These results showed that SIRT1 is a potential promoter in EMs progression. The above results revealed that METTL3 and SIRT1 perform beneficial reverse functions in ESCs. Moreover, we showed that reduced SIRT1 expression exacerbated the repression of METTL3-induced limitations in viability, proliferation, and migration and promoted cellular senescence escape in ESCs, providing further evidence of the mutual interaction. In summary, METTL3 acts as an EMs inhibitor by modulating SIRT1 expression in ESCs.

### METTL3-induced SIRT1 mRNA decay is dependent on m6A-YTHDF2

**Fig. 5:**
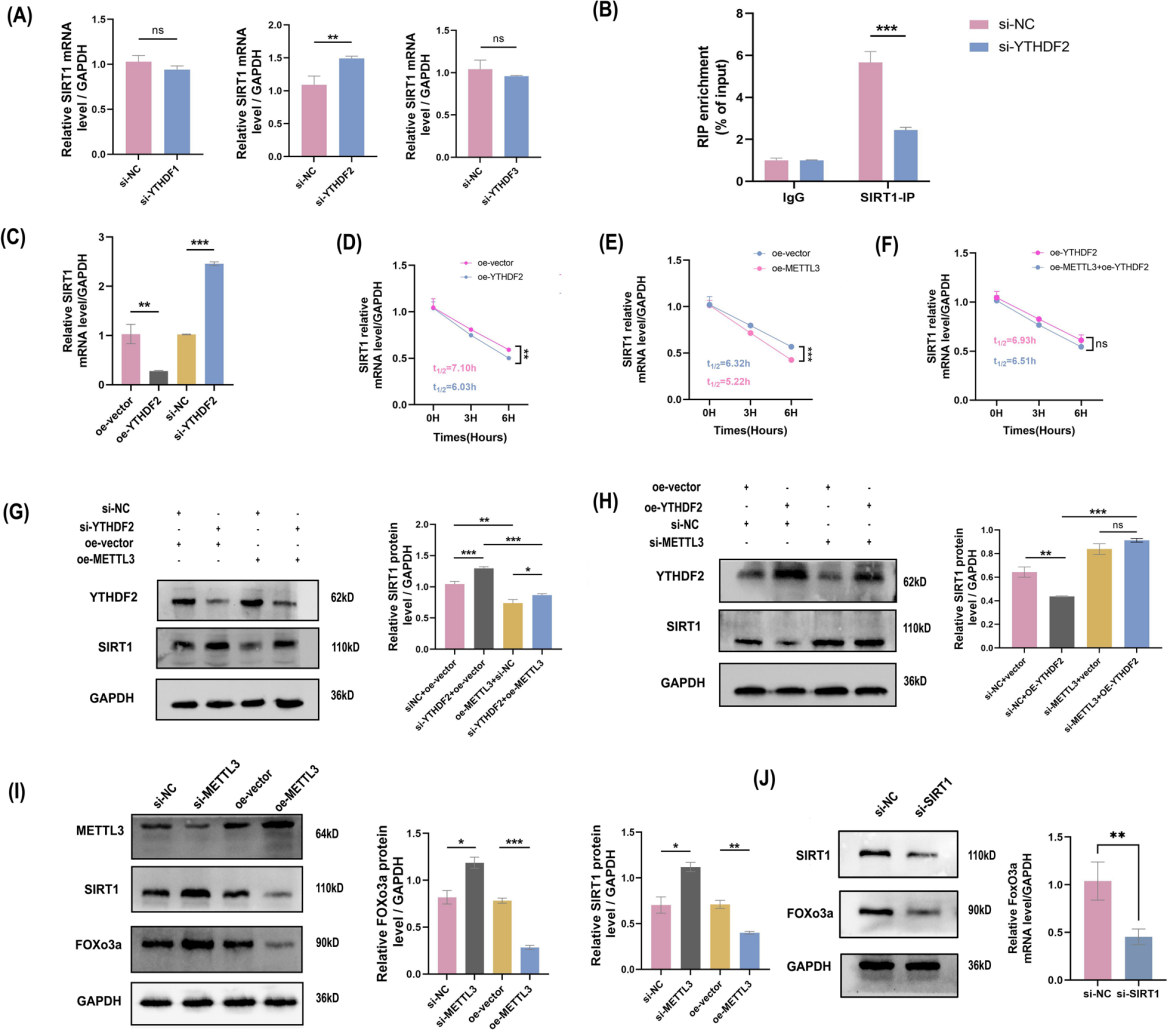
METTL3 reduced the mRNA stability of SIRT1 via YTHDF2 depending on the m6A manner. **A** SIRT1 mRNA level in ESCs detected undergo si-YTHDF1, si-YTHDF2, si-YTHDF3 transfection by RT-qPCR. **B** Reduction enrichment level of SIRT1 in ESCs after YTHDF2 depleting by RIP-qPCR. **C** mRNA level of SIRT1 in YTHDF2 knockdown and overexpression by RT-qPCR. SIRT1 mRNA decay analysis at the indicated times after actinomycin D (Act D, 5 μg/ml) treatment in ESCs under YTHDF2 overexpressed **D**, METTL3 overexpressed **E**, and both overexpressed **F** by RT-qPCR. **G** The protein level of YTHDF2 and SIRT1 in ESCs transfected with si-NC, si-YTHDF2, oe-METTL3, and si-YTHDF2 + oe-METTL3 **H** and si-NC, oe-YTHDF2, si-METTL3, and oe-YTHDF2 + si-METTL3 by western blotting, using GAPDH as an internal control. **I** The protein level of METTL3, SIRT1, and FOXo3a in increasing and exhausting METTL3 treated ESCs. **J** The protein level of SIRT1 and FOXo3a in si-SIRT1 transfected ESCs, using GAPDH as an internal control. All above result were shown in means ± SD, ns, p ≥ 0.05; *p < 0.05; **p < 0.01; ***p < 0.001

m6A modification depends on the functional roles of reader proteins in biological processes. To identify the readers of SIRT1, we measured SIRT1 mRNA levels after reducing YTHDF1, YTHDF2, and YTHDF3 expression. si-YTHDF2 showed the most significantly enhanced ability (Fig. 5A). A quantitative RIP assay was performed to assess the levels of SIRT1 mRNA bound to YTHDF2 in ESCs. SIRT1 enrichment was drastically reduced in ESCs upon the elimination of YTHDF2 expression (Fig. 5B). Then, we found that YTHDF2 knockdown substantially increased SIRT1 expression, and overexpression of YTHDF2 showed a decreased effect (Fig. 5C). Based on previous research, we considered YTHDF2 to act as a m6A reader in the EMs model. YTHDF2 (YTH domain family 2), which is recognized as a m6A reader protein, affects the stability of transcripts. We tested whether m6A modification affects the mRNA stability of SIRT1. We treated ESCs with the transcription inhibitor actinomycin D (Act D), and the half-lives of SIRT1 transcripts were significantly decreased in oe-METTL3 ESCs (Fig. 5D) and oe-YTHDF2 ESCs (Fig. 5E) compared with the control group. Further to confirm the involvement of m6A reader YTHDF2 in the stability regulation of SIRT1 transcripts, we modulated METTL3 and YTHDF2 expression simultaneously in ESCs and examined the SIRT1 mRNA decay rate after Act D treated (Fig. 5F), which given evidence that YTHDF2 influenced the stability of SIRT1 transcripts through m6A modification independently.

However, silencing YTHDF2 led to increased SIRT1 expression, and this effect could be partially repressed by METTL3 overexpression (Fig. 5G). In contrast, the reduction in m6A modification levels induced by the reduction of METTL3 expression led to increased expression of SIRT1, and this effect cannot be remedied in ESCs by upregulation of YTHDF2 expression (Fig. 5H). Our findings indicate that METTL3-mediated m6A modification maintains SIRT1 expression in a m6A-YTHDF2-dependent manner by regulating SIRT1 mRNA stability.

### METTL3 induces ESCs dysfunction by regulating SIRT1-FoxO3a cellular senescence dynamics

**Fig. 6:**
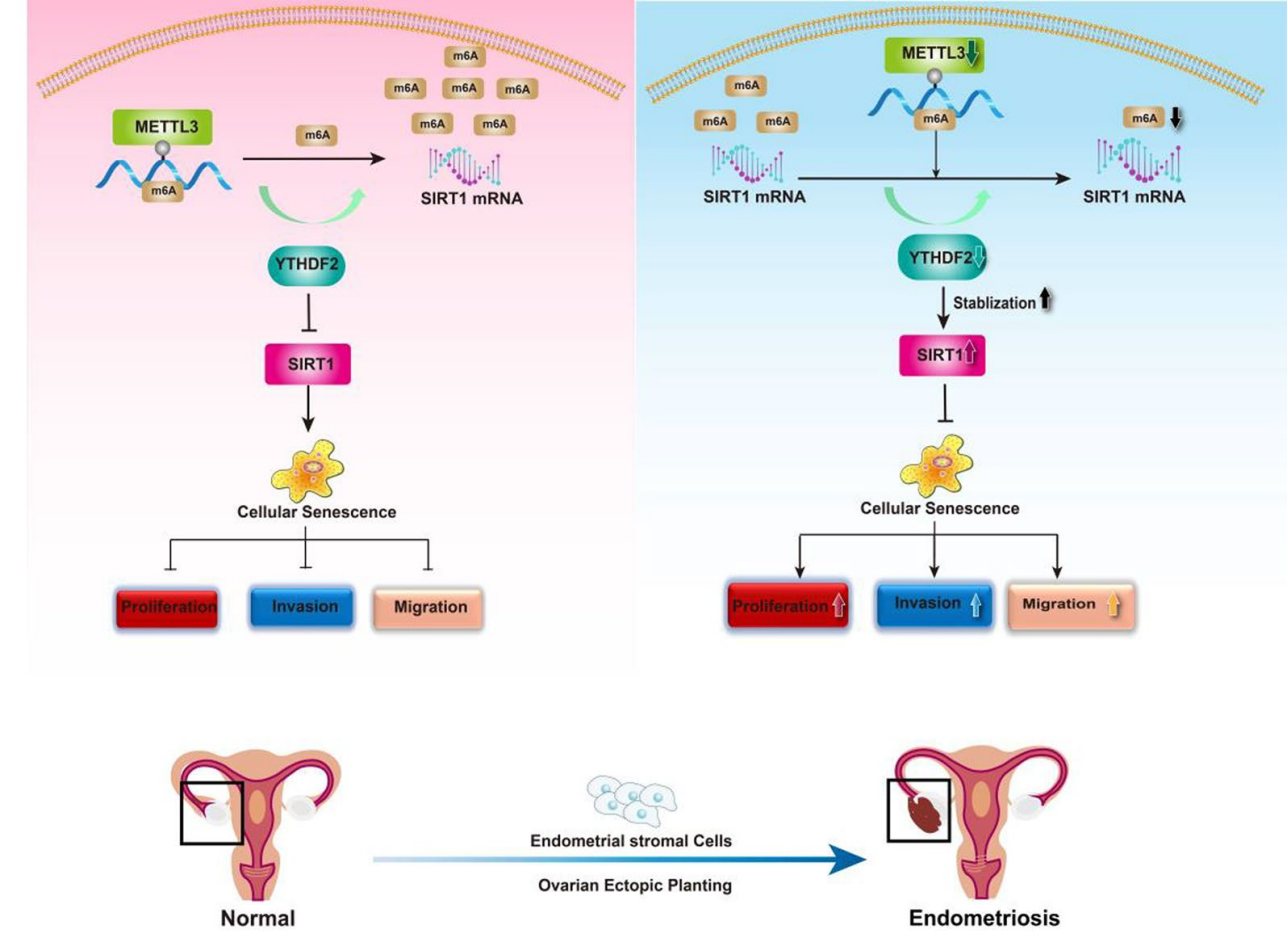
Schematic representation mechanisms of METTL3 inhibit the progression of Endometriosis by regulating SIRT1 through m6A-dependent manner

Previous studies have shown that the SIRT1-FoxO3a signalling pathway is critical in regulating cellular senescence. In the above m6A microarray, the KEGG results showed that an activated FoxO signalling pathway with SIRT1 enrichment may be downstream of METTL3 modification in EMs. Our study demonstrated that SIRT1 is negatively regulated by METTL3 expression. To further explore whether the SIRT1/FOXO3a pathway played a role in METTL3-induced ESC senescence escape in EMs, we pretreated cells with si-METTL3 and oe-METTL3. Western blotting analysis showed that silencing METTL3 expression indeed restored the reductions in SIRT1 and FOXO3a protein levels (Fig. 5I). In addition, a si-SIRT1 ESC model was employed to investigate the positive role of SIRT1 in FOXO3a expression (Fig. 5J). These results suggest that METTL3 reduces m6A modification levels and cellular senescence in ESCs by inactivating the SIRT1/FOXO3a pathway. Finally, we propose a model for the critical link between METTL3-m6A-SIRT1-YTHDF2 in EMs progression (Fig. 6).

## Discussion

Endometriosis (EMs) is one of the most common benign tumours that causes infertility and chronic pelvic inflammation in 20% of bearing-aged women worldwide [33]. EMs is caused by the presence of functional oestrogen-dependent endometrial tissues outside the uterine cavity. It is estimated that a wide range of epigenetic modifications occur in endometriosis [34], resulting in abnormal alterations in the endometrium. The progression of ectopic planting drivers involves several critical epigenetic signalling pathways that promote ESC growth and proliferation as well as migration, adherence, and release of inflammatory factors [35]. These key drivers, including N6-methyladenosine, are studied given their critical roles in the genesis and progression of EMs. Assessment of the roles of epigenetics and the molecular pathogenesis of EMs should aid in the exploration of the pharmacological inhibition of dominant targets.

It is widely accepted that m6A is an abundant biological posttranscriptional modification in eukaryotic mRNAs and ncRNAs [36], affecting the stability of associated genes and leading to tumorigenesis in a broad range of cancer types [37]. The homeostasis of m6A RNA methylation is maintained by adjusting the levels of m6A “writers” and “erasers”. It has been reported that METTL3 [38] and FTO [39] participate the progression of EMs, but their modulatory roles in EMs remain largely unknown. In this study, METTL3 inhibited the advancement of EMs through the enhancement of cellular senescence, which is activated by the SIRT1/FOXO3a signalling pathway. To our knowledge, this is the first study that shows the complete role of m6A-mediated modification in the progression of EMs.

By measuring global m6A levels, we consistently noticed a decreasing trend in ectopic endometrial tissue with decreased expression of “writers” and elevated expression of “erasers”. Considering the fluctuation of m6A RNA levels and the noticeable downregulation of METTL3 expression in EMs compared to eutopic and normal endometrial specimens, the methyltransferase METTL3 was chosen as the target in our study. Furthermore, gradually declining METTL3 expression is related to an increased r-AFS stage, larger cyst size and DIE. In our previous study, METTL3, the major component of the m6A methyltransferase complex [40], demonstrated the ability to function independently and may be a diagnostic target of EMs in the GEO database. According to our study, reducing m6A methylation levels in human ESCs by suppressing METTL3 expression leads to cell proliferation, cell invasion, and migration activation, consistent with previous studies. However, another report indicated that FTO, instead of METTL3, plays a pathogenic role in EMs, suggesting that m6A modification is an evolutionary process and that the dominant shift in m6A exhibits different patterns in human disease.

Cellular senescence has been observed in ESCs, which exhibit acute cellular senescence in normal endometrium for homeostasis maintenance [41] to promote endometrial remodelling at the time of embryo implantation and the menstrual cascade. Previous research showed that EMs were associated with significantly lengthened telomeres and elevated telomerase expression [42]. These processes preserve the telomeres that coat the ends of chromosomes, thereby prolonging the proliferative lifespan of a cell and escaping cellular senescence. Given that METTL3 overexpression had an anti-progression effect, we demonstrated that METTL3 alteration could modulate cellular senescence in ESCs. It was demonstrated that escape from senescence enhances tumour growth, while considering EMs exhibit numerous biological behaviours similar to malignancies, the senescence mechanism could be more convinced in EMs. Consistent with our current study, it could be suggested that senescent ESCs share the characteristics of epigenetically induced senescence, including increased SA-β-Gal activity and the expression of the senescence-related p53-p21 pathway and senescence-related markers (Lamin b1). In our study, the inhibition of METTL3 expression promoted EMs in vivo and in vitro and reduced the expression of senescence promoters. METTL3 overexpression led to the opposite effect. It is widely known that epigenetics is widely involved in the biological process of cellular senescence. The previous study primarily focused on epigenetic senescence in degenerative disease, and this is the first study to reveal a close relationship between cellular senescence and N6-methyladenosine in invasive disease.

To explore METTL3-mediated mRNA m6A modification in EMs, a m6A microarray assay and RNA-seq analysis were performed in METTL3-overexpressing ESCs to identify candidate downstream mRNAs. Among multiple differential expressed and variably methylated mRNAs, SIRT1 was chosen given its role as a classical autophagy, inflammation, and cellular senescence regulator [43]. SIRT1 participates in the endometrial microenvironment for decidualization [44] and exhibits unique expression patterns in endometriosis. Early evidence has demonstrated that SIRT1 inactivation protects mice against implantation failure [45], decidualization defects, and progesterone resistance, suppressing the progression of endometriosis. Our results reveal a increased SIRT1 expression in ectopic endometrial tissue, which is negatively regulated by METTL3, and indicate that the anti-progression effect in EMs induced by METTL3 might be mediated by SIRT1 methylation. To provide robust evidence of the interaction, we constructed a CKO mouse donor-recipient endometriosis model. This model was used to verify the inhibitory role of METTL3 in EMs and provided strong evidence of the negative regulation of METTL3 and SIRT1 at the in vivo level. To precisely assess the modulation of SIRT1 expression by METTL3, we first evaluated SIRT1 expression levels in METTL3 overexpression and deletion cell models and revealed the negative regulation of SIRT1 by METTL3. Second, we performed MeRIP and METTL3-RIP, demonstrating that METTL3 directly interacts with SIRT1 and inhibits SIRT1 expression by increasing SIRT1 m6A modification levels.

Under these circumstances, these assays revealed that METTL3 prevented ESCs migration, invasion, and proliferation via the downregulation of SIRT1, directly preventing the progression of EMs. Importantly, SIRT1 is an essential factor that delays cellular senescence by regulating diverse biological processes through the regulation of senescent factors, such as p53, a cellular senescence promoter, and participate tumour progression through p21 activation; SIRT1 also deacetylates p53-p21 to inhibit biological processes [46]. In our study, inhibition of SIRT1 expression accelerated the ESCs senescence induced by METTL3, and the subsequent decrease in cell proliferation prevented the progression of EMs.m6A reader regulators predominantly recognize epigenetic m6A modifications, and the activity of YTHDF2 has been confirmed to promote the degradation of mRNA [47]. Given that METTL3 negatively impacts SIRT1, and combined with our previous results from bioinformatics [48], we hypothesized that YTHDF2 was a vital ‘reader’ that triggered SIRT1 mRNA decay. METTL3 overexpression induced a reduction in mRNA half-life compared to the control, indicating that YTHDF2 potentially functions as the central ‘reader’ of SIRT1. This notion was further confirmed by YTHDF2-RIP assays. As expected, YTHDF2 reduction remarkably upregulated SIRT1 at the protein level. In contrast, YTHDF2 overexpression cannot reduce the increased SIRT1 levels induced by METTL3 inhibition. These results revealed that METTL3 inactivated SIRT1 via a m6A-YTHDF2-dependent mechanism and pointed to m6A modification as the most crucial link of the complete axis.

According to our m6A epitranscriptomic microarray results, SIRT1 was enriched in both cellular senescence and the FoxO signalling pathway. A previous study widely described the close relationship between cellular senescence and SIRT1/FoxO3a. FoxO3a is a member of the FoxO family, combines with SIRT1, and participates in the cellular senescence process [49]. In the current study, METTL3 upregulation significantly reduced SIRT1 and FoxO3a expression, further demonstrating that METTL3 induces cellular senescence by partially targeting SIRT1/FoxO3a signalling.

Consequently, our observations revealed a novel epigenetic mechanism and provide compelling evidence that METTL3 potentially reduces the stability and enhances cellular senescence function of SIRT1 in a m6A-YTHDF2-dependent manner. Moreover, under conditions of abnormal m6A modification levels, SIRT1 promoted EMs progression, thereby providing a molecular basis for the future use of N6-methyladenosine agonists of SIRT1 mRNA in clinical research. However, some limitations to this study should be noted. First, patients with DIE, a particular type of EMs, shows hyper METTL3 expression compared to other ovarian endometriosis alone specimens. The m6A methylation mechanism needs to be further assessed. Second, verification of the m6A methylation position needs to be investigated at additional levels to demonstrate the primary METTL3-dependent epigenetic regulatory strategy of SIRT1. Third, the effect of the SIRT1/FOXO3a signalling pathway on ESCs senescence should be explored further. These limitations reduce the strength of our conclusions. Ultimately, greater attention should be devoted to future research, as the role of METTL3 and the SIRT1/FOXO3a signalling pathway in EMs requires further clarification.

## Supplementary Information

**Additional file 1: Table S1.** Primers sequences Table.** Table S2.** The sequences of the siRNAs.

## Data Availability

We are ready to provide details regarding this if published.
