# Supplementary material for: METTL3-mediated m6A modification of SIRT1 mRNA inhibits progression of endometriosis by cellular senescence enhancing
Source: J Transl Med. 2023 Jun 23;21:407. doi: 10.1186/s12967-023-04209-0 (PMC10288727; doi:10.1186/s12967-023-04209-0)
Supplement: Supplementary file 1 — Additional file 1: Table S1. Primers sequences Table. Table S2. The sequences of the siRNAs. [file 12967_2023_4209_MOESM1_ESM.doc]

**Additional Materials:**

Table S1: Primers sequences Table

| Gene Name | Primers sequences |
| --- | --- |
| YTHDF2 | Forward:GGTTCTGTGCATCAAAAGGATGG |
|  | Reverse:CCAAAGAATAGGAAAAGCCAATGG |
| METTL3 | Forward:ACCCTGACAGATGATGAGATGC |
|  | Reverse:CGTTCATACCCCCAGAGGTTTAG |
| METTL14 | Forward:GTTGGAACATGGATAGCCGC |
|  | Reverse:CAATGCTGTCGGCACTTTCA |
| WTAP | Forward:GCGAGACCCACAAATAAAGGG |
|  | Reverse:CATCTTGAATCCTCTCCAGGCA |
| FTO | Forward:TGGTGTCCCAAGAAATCGTG |
|  | Reverse:TGCAGGCCGTGAACCAC |
| ALKBH5 | Forward:TCAGCATCGGAACCAGCA AAG |
|  | Reverse:TCCTGACTGACCTTCTTGCTC |
| YTHDF1 | Forward:ATGTCGGCCACCAGCGTGGACA |
|  | Reverse:TCATTGTTTGTTTCGACTCTGC |
| YTHDF2 | Forward:GGTTCTGTGCATCAAAAGGATGG |
|  | Reverse:CCAAAGAATAGGAAAAGCCAATGG |
| GAPDH | Forward:GGAGCGAGATCCCTCCAAAAT |
|  | Reverse:GGCTGTTGTCATACTTCTCATGG |

Table S2: The sequences of the siRNAs

| si-RNA Name | Primers sequences |
| --- | --- |
| si-METTL3 | Forward:CGUCAGUAUCUUGGGCAAG TT |
|  | Reverse:CUUGCCCAAGAUACUGACG TT |
| si-SIRT1 | Forward:CAGGUCAAGGGAUGGUAUUUA TT |
|  | Reverse:UAAAUACCAUCCCUUGACCUG TT |
| si-YTHDF1 | Forward:AGCUAAUCUAGACCAAAGA TT |
|  | Reverse:UCUUUGGUCUAGAUUAGCU TT |
| si-YTHDF2 | Forward:CCGCGUCUAGUUGUUCAUGAA TT |
|  | Reverse:UUCAUGAACAACUAGACGCGG TT |
| si-YTHDF3 | Forward:UUGUCCAGGAUAUAUUUCC TT |
|  | Reverse:TGCAGGCCGTGAACCAC TT |
| si-NC | Forward: UUCUCCGAACGUGUCACGU TT |
|  | Reverse:ACGUGACACGUUCGGAGAA TT |
| GAPDH | Forward:UAAAGUACCCUGUGCUCAA TT |
|  | Reverse:UUGAGCACAGGGUACUUUA TT |
